# A Systematic Review of Cost-Effectiveness of Treating Out of Hospital Cardiac Arrest: Implications for Resource-limited Health Systems

**DOI:** 10.21203/rs.3.rs-4402626/v1

**Published:** 2024-06-04

**Authors:** Kalin Werner, Sarah Hirner, O.Agatha Offorjebe, Edouard Hosten, Julian Gordon, Heike Geduld, Lee A. Wallis, Nicholas Risko

**Affiliations:** University of California, San Francisco; University of Colorado School of Medicine; LAC+USC Department of Emergency Medicine; Université Libre de Bruxelles; East Carolina University; Stellenbosch University; University of Cape Town; Johns Hopkins University School of Medicine

**Keywords:** Emergency care, Cardiac Arrest, Cost-effectiveness, Health Policy, Health Economics, Public Health

## Abstract

**Background:**

Out-of-hospital cardiac arrest (OHCA) is a prevalent condition with high mortality and poor outcomes even in settings where extensive emergency care resources are available. Interventions to address OHCA have had limited success, with survival rates below 10% in national samples of high-income countries. In resource-limited settings, where scarcity requires careful priority setting, more data is needed to determine the optimal allocation of resources.

**Objective:**

To establish the cost-effectiveness of OHCA care and assess the affordability of interventions across income settings.

**Methods:**

The authors conducted a systematic review of economic evaluations on interventions to address OHCA. Included studies were (1) economic evaluations (beyond a simple costing exercise); and (2) assessed an intervention in the chain of survival for OHCA. Article quality was assessed using the CHEERs checklist and data summarised. Findings were reported by major themes identified by the reviewers. Based upon the results of the cost-effectiveness analyses we then conduct an analysis for the progressive realization of the OHCA chain of survival from the perspective of decision-makers facing resource constraints.

**Results:**

468 unique articles were screened, and 46 articles were included for final data abstraction. Studies predominantly used a healthcare sector perspective, modeled for all patients experiencing non-traumatic cardiac OHCA, were based in the US, and presented results in US Dollars. No studies reported results or used model inputs from low-income settings. Progressive realization of the chain of survival could likely begin with investments in TOR protocols, professional prehospital defibrillator use, and CPR training followed by distribution of AEDs in high-density public locations. Finally, other interventions such as indiscriminate defibrillator placement or adrenaline use, would be the lowest priority for early investment.

**Conclusion:**

Our review found no high-quality evidence on the cost-effectiveness of treating OHCA in low-resource settings. Existing evidence can be utilized to develop a roadmap for the development of a cost-effective approach to OHCA care, however further economic evaluations using context-specific data are crucial to accurately inform prioritization of scarce resources within emergency care in these settings.

## INTRODUCTION

Cardiac arrest is a leading cause of death in high-income countries.^1^ Given its sudden and often dramatic presentation, this condition draws significant public attention. Since the introduction of interventions such as cardiopulmonary resuscitation (CPR) and external defibrillation in the mid-20th century, emergency care systems in high-resource settings have devoted substantial effort and funding toward increasing access to treatment for cardiac arrest. A “Chain of Survival” focusing on non-traumatic out-of-hospital cardiac arrest (OHCA) has been conceptualized to include: activation of the emergency care system; bystander/professional CPR; public access/professional defibrillation; medications; transport to the facility; and post-arrest care in cases of return of spontaneous circulation (ROSC).^2^ Bystander training programs, public awareness campaigns, and public placement of automated external defibrillators (AEDs) are examples of public health efforts to improve outcomes.

Despite these innovations, outcomes remain poor across the globe. A 2010 systematic review of 67 international studies examining OHCA found a global survival rate of ~ 7%.^3^ Globally 30-day survival or survival to hospital discharge remains low: 8.8% in a 2020 systematic review.^4^ Even in health systems able to devote ample resources towards the chain of survival, the challenges of addressing OHCA have endured. For example, nationally representative samples or registries from the United States, Japan, and the United Kingdom reported survival to hospital discharge rates ranging from 2.3–8.5%.^5–7^ Among the limited number of survivors, neurologic sequelae are extensive and financially burdensome, and life expectancy is short.^3,8–10^

Over 80% of the world’s population lives in low or middle-income countries (LMICs), but only 20% of global healthcare spending is devoted to this population.^11^ Few of these healthcare dollars go to emergency care systems, despite evidence that over 50% of the disease burden is attributable to conditions amenable to emergency care.^12^ In low-resource settings, where scarcity requires careful priority setting, it is unclear whether the investment in interventions to address OHCA represents the best use of resources. In settings where emergency care systems are in early development, it is critical to evaluate the cost-effectiveness of OHCA interventions to better inform resource allocation which maximizes health benefits.

We conducted a systematic review of the economic evidence surrounding interventions for non-traumatic OHCA in all settings, including specific interventions in the chain of survival. We then use these results and apply them to our knowledge about low-resource settings to better understand the potential costs and effects of OHCAs in low-resource settings. The results will inform ongoing discussions occurring in countries across the world regarding the allocation of scarce resources within emergency care development.

## METHODS

We conducted a systematic review of the literature using the Preferred Reporting Items for Systematic Review and Meta-Analyses (PRISMA) guidelines.^13^ The review was accepted and registered with the International prospective register of systematic reviews (PROSPERO-CRD42022310780). Six databases (PubMed, EMBASE, Global Health, Cochrane, Global Index Medicus, and Tuft’s Cost-Effectiveness Registry) were searched for articles related to the cost-effectiveness of emergency care interventions to treat non-traumatic OHCA. Search terms were entered in English, without language restriction on the article’s language or date restriction. Broad terms that would capture any research around OHCA and its interventions were paired with cost-effectiveness terms. Traumatic cardiac arrest fell outside of the scope of this review.

A sample of PubMed search terms is included in Box 1. Inclusion criteria for an article progressing to data extraction were: (1) An economic evaluation (beyond a simple costing exercise); and (2) an assessment of an intervention in the chain of survival for OHCA.

Following PRISMA guidelines, two reviewers independently screened articles for relevance based on title and abstract. Studies that did not use an economic evaluation study design, or were not related to an OHCA care intervention were excluded. Full texts of the remaining articles were then screened again for inclusion eligibility with any conflicts requiring resolution by a senior reviewer. The reference lists of all included texts were hand-searched for further potential inclusions.

Included studies were then assessed for quality using the Consolidated Health Economic Evaluation Reporting Standards (CHEERS) guidelines.^14^ The statement uses 25 checklist items across six main categories ranging from (1) title and abstract; (2) introduction; (3) methods; (4) results; (5) discussion; and (6) other. Scores of this exercise can be found in Appendix 1. Studies were not excluded from our review based on their scores.

For each study that met all inclusion criteria data were abstracted using a predefined 17-item extraction matrix. To characterize the attributes of included studies, data relevant to economic evaluations were extracted including country of study, intervention and comparator, study design, characteristics of the study population, perspective, time horizon, discount rate, currency and currency year, willingness to pay threshold, and incremental cost-effectiveness ratio. Included studies were first analysed by theme through discussion and consensus amongst reviewers. We then conducted a decision analysis from the perspective of decision-makers with resource limitations.

### Patient and Public Involvement

There was no patient or public involvement in the methods or conduct of this study.

## RESULTS

**Figure 1:**
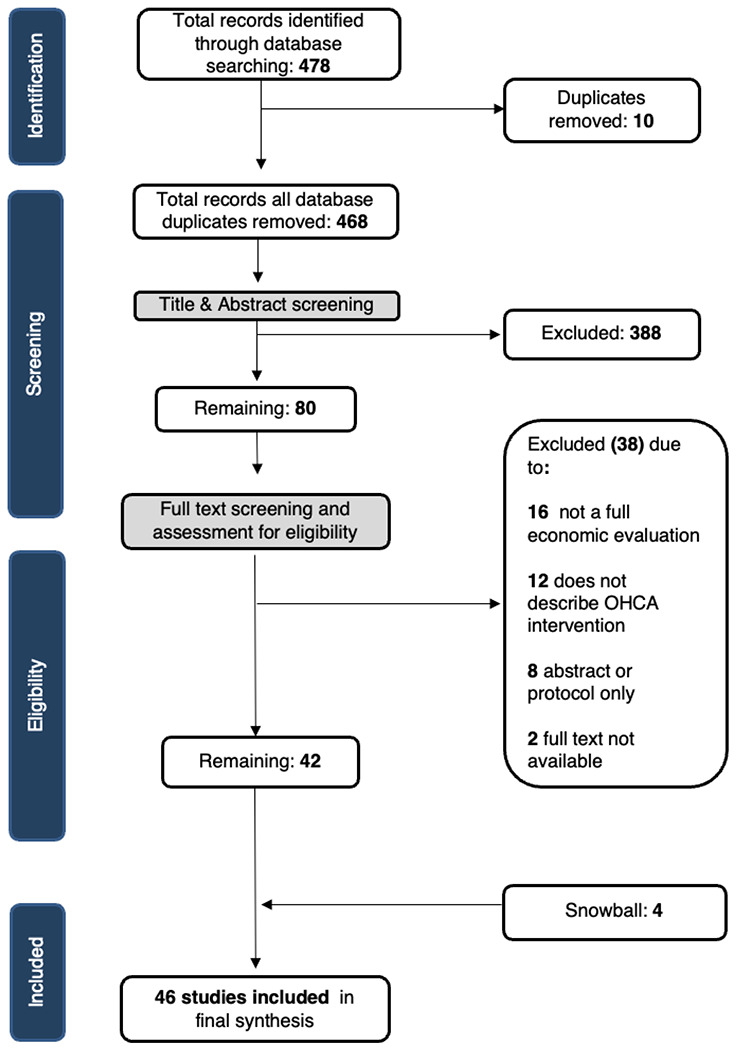
PRISMA Flow Diagram

Figure 1 presents our PRISMA flow diagram. 478 articles were identified in our initial search. After removing ten duplicate studies, 468 studies were assessed on title and abstract. 80 full-text articles were then screened by two blinded independent reviewers. During this phase, 38 studies were excluded. The majority of studies (16) were excluded due to not being full of economic evaluations. Six of the 16 were systematic reviews that fell outside of our inclusion criteria; however, in this case the authors searched the full reference lists of all systematic reviews for additional relevant literature. Eight studies were only available as abstracts, and two studies were unavailable in full text. Twelve studies did not describe OHCA interventions and two studies did not assess a health outcome measure. Reference lists were checked for all relevant studies, leading to the identification of an additional four studies. 46 studies were eligible for data abstraction.

### Study characteristics

Descriptive characteristics were identified in the included studies and are discussed below. Table 1 includes a summary of the abstracted data from the systematic literature review.

### Perspective

The choice of perspective determines which costs and effectiveness outcomes are included in the analysis. Studies in our review used three categories of perspectives including societal, health system/health care sector, and patients/ payors. While a health sector perspective considers only costs incurred in the provision of medical care, the societal perspective accounts for medical costs as well as lost wages and productivity due to ill health. Best practice recommends the use of societal and healthcare sector perspectives.^15,16^ Most studies in our review approached their analysis using a healthcare sector perspective (22 articles: 48%), followed by those that used a societal perspective (12 articles; 26%). A single study used the patient-payer perspective, and only two studies conducted their analyses using both a health system and societal perspective.^17,18^ The remaining studies did not report the perspective used in their analysis (9 articles; 20%).

### Intervention and comparator

Public access automated external defibrillators (AEDs) were the most common intervention assessed (18 articles; 39%). Eight studies (17%) compared CPR and bystander training. Studies focusing on pre-hospital interventions compared various attributes of EMS systems capable of resuscitating patients against those without such a capability^19^ as well as the value of prehospital critical care.^20^ Frequently this focused on comparing the deployment of various cadres of support providers including specially trained emergency medical technicians,^21–23^ police personnel,^24^ and fire stations^25^, to the current standard of only emergency physicians. The effect of reducing ambulance response time was addressed in two studies.^26,27^ The use of adrenaline during resuscitation was compared to saline placebo in two studies.^28,29^ iGel supraglottic airway was assessed compared to tracheal intubation during arrest in two studies.^30,31^

### Simulated population

The age of the simulated population ranged. Most of the included studies did not limit their population to a particular age, modeling for all patients experiencing non-traumatic cardiac OHCA (29 articles; 63%). However, some studies simulated adult-only populations (13 articles; 28%), while only two studies simulated a cohort of children with cardiac conditions.^32,33^ Furthermore, the characteristics of simulated populations were highly heterogeneous. While some studies specified patients by characteristics related to their OHCA, such as patients who were witnessed with a shockable rhythm,^26,34^ others specified patient populations where there was a lack of witness or EMS present at the time of the arrest.^17^ Some studies required patients to be unconscious^35–37^ or that resuscitation had been attempted.^38^

### Country and currency

Eighteen studies were based in the US (39%) and results were presented in US Dollars. Six studies were based outside of the US but converted results from local currency to US Dollars including Canada (2), Sweden (1), Denmark (1), Israel (1), and Taiwan (1). The remaining studies included models where results were presented in the given country’s currency including the United Kingdom (8), Canada (1), Sweden (1), Austria (1), Australia (1) Belgium (1), Colombia (1), Italy (1), Germany (1), Mexico (1), Ireland (1), Qatar(1) the Netherlands (1), Japan (1), and Singapore (1).

### Modeling approach

Studies in our review covered a variety of modeling approaches. Most studies used a decision-analytic model (20 articles; 43%). Ten studies used Markov-based models (including decision-analytical Markov models). One study utilized a microsimulation approach.^39^ However, a fair number of studies did not report on their model approach (9 articles; 20%).

### Time horizon

Time horizons used in the models ranged from six months to a lifetime horizon. Fifteen studies (33%) did not report the time horizon used in their model.

### Discounting

Discount rates ranged from 1.5–6%. The most common rate chosen was 3% (16 articles; 35%). Three studies where the time horizon of the analysis was 1 year or less did not apply discounting. Fifteen studies (33%) did not report the use of a discount rate.

### Threshold used

The cost-effectiveness threshold used for analysis ranged from £20,000 to $150,000/QALY and $19,000/life saved to €65,000/ life saved. The UK National Institute for Health and Care Excellence (NICE) approved threshold of £20,000- £30,000 was the most commonly utilized (8 articles; 17%), closely followed by $100,000 (6 articles; 13%) and $50,000 (5 articles, 11%). Fourteen studies (30%) did not report specific thresholds for their analysis.

### Incremental Cost-Effectiveness Ratios and Other Cost Analysis Findings

Studies focused primarily on CPR training, public or increased access to defibrillation, and prehospital interventions. A single study evaluated the cost-effectiveness of termination of resuscitation (TOR) protocols. A summary of these findings, with currency standardized to 2022 USD, is included in Table 2.

### Quality assessment

Seven articles (15.2%) achieved lower than 50% of the CHEERS list and were considered to be of poor quality. ^22,24,27,35,39–41^ These studies did not report all parameters used in their analysis, nor conducted sensitivity analyses around key variables of uncertainty. However, most studies (25 articles; 54.3%) achieved between above 50–80% of the CHEERs list, and 15 articles (32.6%) could be considered high quality achieving 80% or more of the CHEERS list.

### Thematic Analysis

Studies tended to focus on four themes: (1) CPR and bystander training, (2) public access to automated defibrillators, (3) prehospital emergency care interventions, and (4) termination of resuscitation (TOR).

### CPR and bystander training

Eight studies on bystander training demonstrated a wide range of cost-effectiveness, from $20,534 to $298,727 USD (2022)/QALY gained. Training laypersons was explored through two studies.^43,45^ Two compared the cost-effectiveness of CPR combined with AEDs against basic CPR^37,47^ while one investigated the use of mechanical CPR versus manual chest compressions.^46^ The results were dependent on factors such as equipment costs (the use of mannequins and AED trainers) and training venues (small size vs mass training). The ICER was highest in the case of trainees living with high-risk individuals over the age of 75; $3,674,607/QALY.^45^

### Public Access Defibrillation

Eighteen studies were identified that presented data related to public access defibrillation. Often public access AEDs were compared against the current standard which was no AEDs, however, in one case, the use of AEDs by laypersons was compared to the use of AEDs by EMS personnel.^64^ Three studies targeted the placement of AEDs in residences of high-risk populations including adults over 60, long-term care facilities, and children with heart conditions.^32,50,52^ Two studies analysed the use of drone-assisted AED networks.^39,48^ Cost-effectiveness ranged from $11,398 (for school-based AEDs) to over $2.4 million USD (2022)/QALY gained (in all private residences), with the results heavily dependent on how well the distribution of defibrillators correlated with the population density of OHCAs. Distributing AEDs using drone networks was assessed in two studies with results of $11,584/QALY to $27,749 USD (2022)/ life year saved.

### Prehospital system interventions

Nineteen studies focused on prehospital care centred around care delivered at the scene or enroute to facilities. Three studies addressed efforts to increase the availability or response times of ambulances equipped with defibrillators and demonstrated a range of cost-effectiveness from $17,275 to $102,260 USD (2022)/QALY gained. A subsection of studies addressed specific pre-hospital interventions. Two of these studies demonstrated ratios of $114,824 to $122,769 USD (2022)/QALY gained for the use of adrenaline. Results were dependent on the selected time horizon; for example Perkins et al 2021 found an ICER of $2,397,888 USD (2022)/QALY over the first 6 months after cardiac arrest, however, this reduces to $114,824/QALY over a lifetime horizon. However, clinical efficacy studies of ACLS medication have had equivocal results, making cost-effectiveness unlikely.^28,29^ Two papers compared igel supraglottic airway (SGA) to tracheal intubation, however, one did not find any evidence of a difference in cost-effectiveness, and another found that igel SGA was less effective and more costly than tracheal intubation.^31^

A single study was identified in our review on the economic value of TOR protocols.^63^ When comparing BLS with TOR to a situation with no TOR, it was determined that the no TOR rule scenario was not cost-effective. Among three scenarios (BLS with TOR, ALS with TOR and no TOR) the BLS with TOR protocols was most cost-effective with an ICER of $23,851/QALY. Given the function of TOR as an administrative tool to reduce resource waste, it can be hypothesized that they would be cost-effective or cost-saving in most settings.^65^

We use the results identified in our review to develop a resource prioritization league table based on the likely cost-effectiveness of a progressive realization of interventions in the chain of survival. Figure 1 illustrates the league table illustrating which interventions could be considered appropriate for prioritized funding. The guide comprises a list of interventions in ascending order (from low to high) based on ICER. This represents a prioritized list of interventions, from high priority (low cost per QALY) to low priority (high cost per QALY) for the aim of generating as many QALYs as possible.

## DISCUSSION

This systematic review described and summarized the published evidence related to the cost-effectiveness of non-traumatic OHCA interventions. We highlight studies that identify cost-effective interventions across four areas of the chain of survival including (1) the use of automated external defibrillators, (2) CPR and bystander training, (3) prehospital system interventions, and (4) termination of resuscitation guidelines. Studies predominantly used a healthcare sector perspective, modeled for all patients experiencing non-traumatic cardiac OHCA, were based in the US, and presented results in US Dollars. No studies reported results or used model inputs from low-income settings.

Using the reported ICERs identified in our review, we develop prioritization recommendations to rank the most valuable elements in the chain of survival based on the generation of QALYs per investment. This is particularly useful for decision-makers working under resource constraints. OHCA interventions ranked from highest to lowest priority include TOR protocols, AED for EMS, improvements to prehospital systems, CPR bystander training, and lastly public access AED placement. Three studies comparing public access versus targeted in-home AED placement under the same context and setting determined targeted placement of AEDs with high-risk patients to be more cost-effective than indiscriminate public access deployment.^32,49,56^

Health economics evaluations, typically represented in the form of cost-effectiveness studies, provide a quantitative tool to help inform resource allocation. While economics should never be the sole driver behind policy or resource decisions, comparing two or more interventions on the basis of costs and effects plays a critical role in priority-setting activities. Should decision-makers be faced with a choice of which of the interventions to adopt first, given limited resources, evidence suggests certain parts of the chain of survival may yield higher health returns for finite resources. Increased public access to AEDs is likely cost-effective, but only when placed in high-density public locations. Defibrillator use by professionals in the prehospital system is likely cost-effective as well. The addition of bystander training in CPR and automated external defibrillators (AEDs) is likely cost-effective for fewer contexts.

If we were to apply the findings of this review to lower resource settings, using the World Health Organization’s definition of cost-effectiveness for health interventions (less than three times the annual gross domestic product (GDP) per capita of a country for each disability-adjusted life year (DALY) averted or quality-adjusted life-year (QALY) saved),^26,27^ for the purpose of health system planning, a majority of the interventions identified in our review would not be recommended in low and lower-middle income countries, with many interventions only being recommended in the high-income setting. It is likely that resources would yield a greater impact if directed towards alternate health interventions, in situations where mutually exclusive allocation decisions must be made in the short term.

Cost-effectiveness evidence does exist for numerous emergency care interventions in low-resource settings.^66^ For example, training lay first responders in basic trauma care in urban Uganda costs $25-$75 USD/life year saved,^67^ prehospital electrocardiograms for patients with acute chest pain in India costs $12.65 USD per QALY gained^68^ and basic paediatric emergency care training and triage costs $148 USD per death averted.^69^ Given the high mortality and morbidity, even when maximal resources are leveraged, questions have been raised regarding the ethics and appropriateness of prioritizing OHCA, particularly in low-resource settings.^12^ The opportunity cost of delaying the implementation of competing interventions that are less costly but more efficacious becomes a concern under tight fiscal constraints.

Progressive realization of the chain of survival could likely begin with investments in TOR protocols, professional prehospital defibrillator use, and CPR training, followed by distribution of AEDs in high-density public locations. Finally, other interventions such as indiscriminate defibrillator placement, or adrenaline use would be the lowest priority for early investment.

Unfortunately, our review yielded only two studies on the OHCA chain of survival in an middle-income setting, both in upper-middle-income settings (Colombia and Mexico). No evidence was found from the most resource-limited settings, hindering the transferability of the results, and highlighting the acute need for more research to be conducted in low-resource contexts. Caution should be taken in extrapolating results from high-income to low-income settings for a variety of reasons including differences in health systems, the values of parameters, and cost-effectiveness thresholds among these settings. Relying on results representative of high-income country (HIC) data also means that some costs, particularly human resources, are inflated compared to a low or middle income (LMIC) setting. However, given the more established emergency care infrastructure in HICs, interventions may perform better and achieve greater outcomes than they would in LMIC emergency care naïive settings as well. Given the lack of transferability of these findings to other settings and the context-specific challenges, more primary research is needed for OHCA in low-resourced settings.

There are several other limitations important to consider for decision-makers wishing to utilize this evidence. For one, publication bias may favour studies with positive results, therefore our analyses may overestimate the benefit of the themes identified in our review. Furthermore, while many studies attempted to isolate the effect of certain elements of the chain of survival, all studies occurred in the presence of pre-existing capacities that may have influenced outcomes or led to an underestimation of costs. For example, studies on the cost-effectiveness of bystander CPR in a high-income country make no attempts to control for the existing ambulance and hospital resources that exist in the status-quo base-case comparator.

Care must also be taken in comparing cost-effectiveness ratios across a wide variety of models or intervention intensities. For example, the cost-effectiveness of public access defibrillation varied significantly based on how targeted the placement of AEDs was, and the cost-effectiveness of prehospital interventions varied widely based on urban versus rural population distribution. Furthermore, the interventions identified in our review are highly heterogeneous and presented incomplete methods in terms of time horizon, modeling approach, and perspective used. Finally, we do not recommend that economic considerations serve as the sole factor behind priority setting, resource allocation, or policy development.

## CONCLUSION

OHCA is a prevalent condition with high mortality and poor outcomes even in settings where extensive resources are devoted to this condition. In settings of resource scarcity where allocation decisions inevitably lead to healthcare rationing, economic analyses can aid prioritization. Our systematic review found 46 studies evaluating the cost-effectiveness of treating non-traumatic OHCA. Our review found no high-quality evidence on the cost-effectiveness of treating OHCA in resource-limited settings. Existing evidence can be utilised to develop a roadmap for the development of a cost-effective approach to OHCA care. However further economic evaluations should be completed using context-specific data to help accurately inform prioritization and resource allocation decisions in these settings.

## Figures and Tables

**Figure 2:**
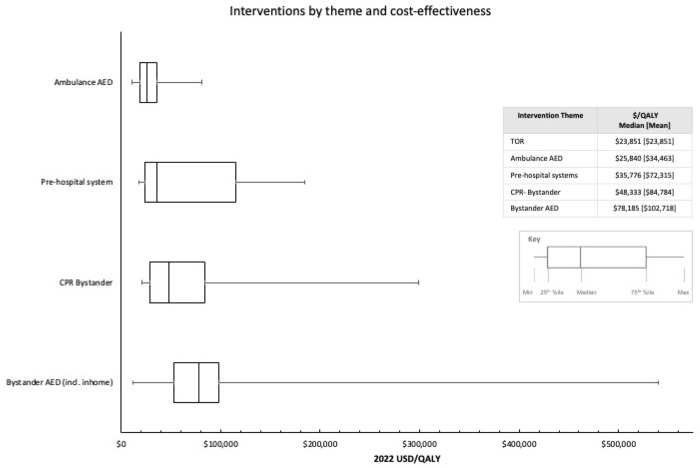
Interventions by theme and cost-effectiveness Note: Selective AED placement includes AEDs distributed by drone networks, and placed in high-risk homes. Only studies reporting ICER outcomes in cost per QALY are included in this table.

**Table 1:** Extracted items from included studies

Study	Study Characteristics		Results
	Descriptive characteristics	Technical characteristics	
Achana (2020)	**Perspective**: Health system **Intervention/Comparator**: Parenteral adrenaline/saline placebo **Simulated population**: 8,014 adult patients with out-of-hospital cardiac arrest in the UK **Country/Currency (adj. year)**: UK;GBP (2017)	**Modeling approach**: Markov model **Time horizon**: 6 months and lifetime **Discounting**: 3.5% **Threshold used**: £20,000 to £30,000/QALY	**Results**: £81,070/QALY **Author’s conclusions**: Adrenaline was not cost-effective when only directly related costs and consequences are considered. However, incorporating the indirect economic effects associated with transplanted organs substantially alters cost-effectiveness, suggesting decision-makers should consider the complexity of direct and indirect economic impacts of adrenaline. **% EE assessment satisfied**: 92%
Aguilera Campos (2012)	**Perspective**: Not reported **Intervention/Comparator**: Increasing number of ambulances vs. keeping same number of ambulances and equipping police with AED and CPR training. **Simulated population**: 11,000 patients with OHCA **Country/Currency (adj. year)**: Mexico;MXN (NR)	**Modeling approach**: Decision-analytic model **Time horizon**: not reported **Discounting**: n/a **Threshold used**: Not reported	**Results**: 5.8–60 million MEX$ /LS ambulance; 0.5–5.5 million meX$ / life saved (police first) **Author’s conclusions**: In Queretaro interventions can be performed taking advantage of the response capacity of the existing police focused on diminishing mortality from OCHA at a lesser cost than delegating this function only to ambulances. **% EE assessment satisfied**: 40%
Al-Badriyeh (2022)	**Perspective**: Payer **Intervention/Comparator**: Out-of-hospital extracorporeal cardiopulmonary resuscitation (OH-ECPR) vs in-hospital extracorporeal cardiopulmonary resuscitation (IH-ECPR) **Simulated population**: 1000 hypothetical OHCA patients with refractory VF or pulseless VT **Country/Currency (adj. year)**: Qatar;QAR (2022)	**Modeling approach**: Decision-analytic model **Time horizon**: 1 year **Discounting**: No discount **Threshold used**: QAR 546,000/case of success	**Results**: $127,634/case of success **Author’s conclusions**: OHECPR for OHCA patients, who are refractory to conventional CPR, is most likely a cost-effective approach relative to the IH-ECPR, supporting the increased utilization of the former as a potentially advantageous resuscitative approach in the OHCA patients. **% EE assessment satisfied**: 88%
Andersen (2019)	**Perspective**: Health system and societal **Intervention/Comparator**: public AED/none **Simulated population**: Public out-of-hospital cardiac arrest in the United States without emergency medicine service personnel present at the time of cardiac arrest. **Country/Currency (adj. year)**: USA;USD (2017)	**Modeling approach**: Decision-analytic markov model **Time horizon**: Lifetime **Discounting**: 3% **Threshold used**: $150,000/QALY	**Results**: $13,700/QALY (health system perspective) or $53,797/QALY (societal perspective) **Author’s conclusions**: Public AEDs are a cost-effective public health intervention in the United States. **% EE assessment satisfied**: 92%
Bauer (2021)	**Perspective**: Not reported **Intervention/Comparator**: Three UAV networks providing 80%, 90% or 100% coverage for rural areas lacking timely access to EMS **Simulated population**: Population living in urban clusters and in urban centres13 and (2) not covered by EMS within 10min time-to-defibrillation. **Country/Currency (adj. year)**: Germany;EUR (NR)	**Modeling approach**: Microsimulation model **Time horizon**: Not reported **Discounting**: n/a **Threshold used**: £20,000 to £30,000/QALY	**Results**: €23,568/LYS **Author’s conclusions**: Demonstrated the relevant life-saving potential of UAV equipped with AED in out-of- hospital cardiovascular arrests: 1477 to 1845 additional years of life can be gained on an annual average compared with EMS. **% EE assessment satisfied**: 36%
Benger (2022)	**Perspective**: Health system **Intervention/Comparator**: i-gel vs tracheal intubation **Simulated population**: Patients aged ≥18 years who had a non-traumatic out-of-hospital cardiac arrest and were attended by a participating paramedic were enrolled automatically under a waiver of consent between June 2015 and August 2017 **Country/Currency (adj. year)**: England;GBP (2018)	**Modeling approach**: within trial economic analysis **Time horizon**: 6 month **Discounting**: No discount **Threshold used**: NR	**Results**: Dominated **Author’s conclusions**: i-gel has a low probability of being cost-effective, regardless of the willingness-to-pay threshold. Overall, there is no evidence of a difference in cost-effectiveness between the groups. **% EE assessment satisfied**: 72%
Benson (2022)	**Perspective**: Societal **Intervention/Comparator**: School-based AEDs vs no AED usage Simulated population: School-based OHCAs in region served by West Midlands Ambulance Service **Country/Currency (adj. year)**: England;GBP (2019)	**Modeling approach**: Decision-analytic model **Time horizon**: Lifetime **Discounting**: No reported **Threshold used**: £20,000/QALY	**Results**: £8,916/QALY **Author’s conclusions**: A strategy of placing AEDs in schools is likely to be cost-effective. **% EE assessment satisfied**: 76%
Bogle (2019)	**Perspective**: Payer **Intervention/Comparator**: AED drone network vs no drone network for AED **Simulated population**: 16,503 OHCAs **Country/Currency (adj. year)**: USA;USD (NR)	**Modeling approach**: Integer linear programming decision model **Time horizon**: 4 yrs **Discounting**: 3% **Threshold used**: $50,000/QALY	**Results**: $858/QALY **Author’s conclusions**: With proper integration into existing systems, large-scale networks for drone AED delivery have the potential to substantially improve OHCA survival rates while remaining cost-effective. **% EE assessment satisfied**: 68%
Bouland (2015)	**Perspective**: Health system **Intervention/Comparator**: Bystander CPR vs no bystander CPR **Simulated population**: 371 nontraumatic OHCAs **Country/Currency (adj. year)**: USA;USD (2013)	**Modeling approach**: Decision-analytic model **Time horizon**: 10 yrs **Discounting**: n/a **Threshold used**: $100,000/QALY	**Results**: $22,539/QALY Author’s conclusions: The cost-effectiveness findings of this analysis demonstrate the financial utility of bystander CPR training. **% EE assessment satisfied**: 88%
Cappato (2006)	**Perspective**: Health system Intervention/Comparator: with and without AED program (AED added to ambulance system) **Simulated population**: Consecutive patients who, outside of the county hospitals, had been unconscious and unresponsive either suddenly or after a brief prodrome, had no palpable pulse, and had no spontaneous respiration. **Country/Currency (adj. year)**: Italy;EUR (NR)	**Modeling approach**: Within trial economic analysis **Time horizon**: Not reported Discounting: n/a Threshold used: Not reported	**Results**: $23,661/QALY Author’s conclusions: UAV equipped with AED can be considered cost-effective and life-saving and can gain 1477 to 1845 additional years of life on an annual average compared with EMS **% EE assessment satisfied**: 36%
Cram (2003)	**Perspective**: Societal **Intervention/Comparator**: Strategy 1: individuals experiencing cardiac arrest were treated by EMS equipped with AEDs, Strategy 2: individuals treated with AEDs deployed as part of a public access defibrination program. -- Public access AEDs **Simulated population**: Simulated cohort of the American public **Country/Currency (adj. year)**: USA;USD (2002)	**Modeling approach**: Decision-analytic model **Time horizon**: Not reported **Discounting**: 3% **Threshold used**: $50,000/QALY	**Results**: $30,000/QALY **Author’s conclusions**: Findings support a policy of AED deployment at selected public locations–and also suggests that deployment of AEDs in hotels and retail stores may not be justified on clinical and economic grounds alone. **% EE assessment satisfied**: 84%
Cram (2005)	**Perspective**: Societal **Intervention/Comparator**: in-home cardiac arrest treated with emergency medical services equipped with AEDs vs received initial treatment with an in-home AED, followed by EMS -- In home AEDs to adults > 60 **Simulated population**: American adults 60 years of age at progressively greater risk for SCD **Country/Currency (adj. year)**: USA;USD (2004)	**Modeling approach**: Markov model **Time horizon**: Lifetime **Discounting**: 3% **Threshold used**: $50,000 to $100,000/QALY	**Results**: $216,000/QALY **Author’s conclusions**: The cost-effectiveness of in-home AEDs is intimately linked to individuals’ risk of SCD **% EE assessment satisfied**: 80%
Doan (2022)	**Perspective**: Health system **Intervention/Comparator**: Extracorporeal cardiopulmonary resuscitation (E-CPR) v conventional CPR (C-CPR) **Simulated population**: **Country/Currency (adj. year)**: Australia;AUD (2021)	**Modeling approach**: Decision-analytic markov model **Time horizon**: Lifetime **Discounting**: 3% **Threshold used**: 82,599 AUD/QALY	**Results**: 45,716 AUD/QALY **Author’s conclusions**: E-CPR has median ICER that is below common accepted WTP thresholds. **% EE assessment satisfied**: 88%
Folke (2009)	**Perspective**: Payer **Intervention/Comparator**: AED vs non AED– Public Access AEDs **Simulated population**: All patients with sudden cardiac arrest confirmed by the absence of consciousness, pulse, and breathing from 1994 to 205–5420 total. **Country/Currency (adj. year)**: Denmark;USD (2008)	**Modeling approach**: Not reported **Time horizon**: 10 yrs **Discounting**: n/a **Threshold used**: Not reported	**Results**: $33,100/QALY (ERC) or $40,900/QALY (AHA) **Author’s conclusions**: A high proportion of cardiac arrests in public can be covered by strategic placement of AEDs within a limited area of a city center and with acceptable costs. **% EE assessment satisfied**: 60%
Forrer (2002)	**Perspective**: Community **Intervention/Comparator**: (1) police first response and ALS care (No-AED) and; (2) AED equipped police first response (P-AED) with subsequent ALS care. **Simulated population**: All adult patients who experienced a cardiac arrest of presumed cardiac origin and were transported to, or received medical direction from William Beaumont Hospital. **Country/Currency (adj. year)**: USA;USD (1999)	**Modeling approach**: Before and after quasi-experimental study **Time horizon**: 7 yrs **Discounting**: n/a **Threshold used**: $19,000/ life saved	**Results**: $50,641/LS or $11,562/LYS **Author’s conclusions**: This study concluded that police could effectively lower the call-to-shock time significantly, and increase ROSC significantly over controls. **% EE assessment satisfied**: 72%
Foutz (2000)	**Perspective**: Health system **Intervention/Comparator**: Placing AEDs in LTCF vs current standard (no AEDs) **Simulated population**: 160 patients in cardiac arrest in long term care facilities **Country/Currency (adj. year)**: USA;USD (NR)	**Modeling approach**: Not reported **Time horizon**: 4 yrs **Discounting**: 5% **Threshold used**: $100,000/QALY	**Results**: $87,837/ LS **Author’s conclusions**: Placing AEDs in LTCFs has a reasonable cost–utility if a hospital discharge survival rate of 25% of patients found in VF can be achieved. **% EE assessment satisfied**: 56%
Ginsberg (2015)	**Perspective**: Societal **Intervention/Comparator**: The “treatment bundle” provided to patients suffering cardiac arrest including basic and advanced life support by the MDA, transfer to an Emergency Medicine Department (ED), treatment in the ED and throughout the hospital admission and appropriate post hospital discharge care (including cardiac rehabilitation, home care, nursing care and mechanical ventilation). **Simulated population**: Patients aged over 18 years with non-traumatic cardiac arrest in the Jerusalem district with EMS documented pulselessness accompanied by a non-perfusing rhythm **Country/Currency (adj. year)**: Israel;USD (2011)	**Modeling approach**: Decision-analytic model **Time horizon**: NR **Discounting**: 3% Threshold used: $87,450/ DALY	**Results**: $28,864/ DALY **Author’s conclusions**: The current package of OHCA interventions in Jerusalem appears to be very cost-effective as the cost per averted DALY of $28,864 is less than the Gross Domestic Product per capita ($33,261). **% EE assessment satisfied**: 60%
Groeneveld (2001)	**Perspective**: Societal **Intervention/Comparator**: AED placement **Simulated population**: 627,956 American Airlines flights in 1997–1999. **Country/Currency (adj. year)**: USA;USD (1997–1999)	**Modeling approach**: Decision-analytic model **Time horizon**: Lifetime **Discounting**: 3% **Threshold used**: $50,000/QALY	**Results**: $35,300 on aircraft > 200 seats; $640,800 on aircraft > 100 seats; $94,700/QALY on all aircraft **Author’s conclusions**: The cost-effectiveness of placing AEDs on commercial aircrafts compares favorable with the cost-effectiveness of widely accepted medical interventions and health policy regulations, but is critically dependent on the passenger capacity of the aircraft. **% EE assessment satisfied**: 88%
Groeneveld (2005)	**Perspective**: Societal **Intervention/Comparator**: three strategies for training a cohort of laypersons in resus citation and defibrillation. (1) training unselected laypersons with a standard CPR/defibrillation course (2) CPR/defibrillation training with purchase of an automated external defibrillator. The final strategy was no CPR/defibrillation training for unselected laypersons. Simulated population: not applicable **Country/Currency (adj. year)**: USA;USD (2004)	**Modeling approach**: Decision-analytic model **Time horizon**: Lifetime **Discounting**: 3% **Threshold used**: Not reported	**Results**: $202,400/QALY general population; $58,800/QALY if trainee lives w/ person > 75 **Author’s conclusions**: Purchase of a home defibrillator was cost-effective at a threshold of $100,000 per QALY if the device cost less than $5, or if the household risk of cardiac arrest exceeded 32 times the national aver- age. None of the sensitivity analyses for unselected training yielded a cost per QALY value $50,000. **% EE assessment satisfied**: 76%
Haag (2020)	**Perspective**: Societal **Intervention/Comparator**: In-home AED **Simulated population**: A theoretical cohort of 15,50 ten-year-old children with hypertrophic cardiomyopathy **Country/Currency (adj. year)**: USA;USD (2019)	**Modeling approach**: Markov model **Time horizon**: Not reported **Discounting**: 3% **Threshold used**: $100,000/QALY	**Results**: $86,458/QALY **Author’s conclusions**: For children at intermediate risk for SCD and HCM, in home AED is cost-effective, resulting in fewer deaths and increased QALYS for a cost below the willingness-to-pay threshold. **% EE assessment satisfied**: 76%
Jakobsson et al (1987)	**Perspective**: Not reported **Intervention/Comparator**: Specially trained EMTs **Simulated population**: 28 patients successfully resuscitated **Country/Currency (adj. year)**: Sweden;USD (1987)	**Modeling approach**: Decision-analytic model **Time horizon**: 1 yr **Discounting**: n/a **Threshold used**: Not reported	**Results**: $14,700/LS **Author’s conclusions**: Training of EMTs is an inexpensive way of providing early defibrillation to out-of-hospital CA patients. **% EE assessment satisfied**: 44%
Jermyn BD. (2000)	**Perspective**: Not reported **Intervention/Comparator**: recently initiated first-responder program in an urban center in southwestern Ontario. **Simulated population**: 88000 **Country/Currency (adj. year)**: Canada;USD (2000)	**Modeling approach**: Not reported **Time horizon**: Not reported **Discounting**: 5% **Threshold used**: Not reported	**Results**: $6,776/LS (urban; control), $49,274/LS (rural; experimental) **Author’s conclusions**: The cost per life saved for a rural first-responder defibrillation program is significantly more expensive than one for an urban center. However, the cost per life saved is still economical compared with common treatments for other life-threatening illnesses. **% EE assessment satisfied**: 52%
Marti (2017)	**Perspective**: Health system **Intervention/Comparator**: LUCAS-2, a mechanical device for CPR as compared to manual chest compressions in adults -- (Mechanical CPR) **Simulated population**: 4,471 adults with non-traumatic, out-of-hospital cardiac arrest from four UK Ambulance Services **Country/Currency (adj. year)**: UK;GBP (Not reported)	**Modeling approach**: Decision-analytic model **Time horizon**: Not reported **Discounting**: n/a **Threshold used**: £20,000/ QALY	**Results**: Manual CPR dominates mechanical CPR **Author’s conclusions**: LUCAS-2 is dominated by manual chest compression **% EE assessment satisfied**: 68%
Mears (2006)	**Perspective**: Not reported **Intervention/Comparator**: AED placement in rural areas **Simulated population**: not applicable **Country/Currency (adj. year)**: USA;USD (NR)	**Modeling approach**: Not reported **Time horizon**: Not reported **Discounting**: n/a **Threshold used**: Not reported	**Results**: $11,457/LS or $2,616/LYS **Author’s conclusions**: This model provides guidance to federal, state, and local EMS administrators who are attempting to identify where to place a limited number of AEDs across a very large geographic area with varying population densities and cardiac arrest rates. **% EE assessment satisfied**: 24%
Moran (2015)	**Perspective**: Societal **Intervention/Comparator**: AED placement **Simulated population**: All EMS attended OHCAs where resuscitation was attempted **Country/Currency (adj. year)**: Ireland;EUR (NR)	**Modeling approach**: Decision-analytic markov model **Time horizon**: Not reported **Discounting**: n/a **Threshold used**: €45,000/QALY	**Results**: €95,640/QALY **Author’s conclusions**: A 40% increase in AED utilisation when OHCAs occur in a public area could potentially render this programme cost effective. **% EE assessment satisfied**: 72%
Nichol (1996)	**Perspective**: Societal **Intervention/Comparator**: (1) improvement in response time by addition of unit hours in a one-tier EMS system by the addition of more EMS providers in ambulances, (2) improvement in response time within a two-tier EMS system by the addition of more BLS/BLS-D providers in pump vehicles to the first tier, (3) improvement in response time within a two-tier EMS system by the addition of more BLS/BLS-D providers in ambulances to the first tier, (4) change from a one-tier EMS system to a two-tier EMS system by addition of BLS/BLS-D providers in pump vehicles as the first tier, (5) change from a one-tier EMS system to a two-tier EMS system by the addition of BLS/BLS-D providers in ambulances as the first tier. **Simulated population**: Cardiac arrest patients **Country/Currency (adj. year)**: Canada;USD (1993)	**Modeling approach**: Decision-analytic model **Time horizon**: Not reported **Discounting**: 5% **Threshold used**: $60,000/QALY	**Results**: $53,000/QALY **Author’s conclusions**: The most attractive options in terms of incremental cost-effectiveness were improved response time in a two-tier EMS system or change from a one-tier to a two-tier EMS system. **% EE assessment satisfied**: 68%
Nichol (1998)	**Perspective**: Health system **Intervention/Comparator**: Standard EMS systems vs EMS supplemented by public access defibrillation (PAD) **Simulated population**: not explicitly mentioned **Country/Currency (adj. year)**: USA;USD (1996)	**Modeling approach**: Decision-analytic model **Time horizon**: Not reported **Discounting**: 3% **Threshold used**: $50,000/QALY	**Results**: $44,000/QALY **Author’s conclusions**: Although more expensive than standard EMS for sudden cardiac arrest, PAD may be economically attractive. **% EE assessment satisfied**: 72%
Nichol (2003)	**Perspective**: Societal **Intervention/Comparator**: Standard EMS versus targeted non-traditional responders **Simulated population**: 148 patients **Country/Currency (adj. year)**: USA;USD (2003)	**Modeling approach**: Markov model **Time horizon**: Lifetime **Discounting**: 3% **Threshold used**: $100,000/QALY	**Results**: $56,700/QALY **Author’s conclusions**: Where cardiac arrest is frequent and response time intervals are short, rapid defibrillation by targeted non-traditional responders may be a good value for the money compared with standard EMS. **% EE assessment satisfied**: 72%
Nichol (2009)	**Perspective**: Societal **Intervention/Comparator**: Public access defibrillation (CPR only vs CPR + AED (bystanders)0 **Simulated population**: Not applicable **Country/Currency (adj. year)**: USA;USD (1996)	**Modeling approach**: Decision-analytic model **Time horizon**: Lifetime **Discounting**: 3% **Threshold used**: $100,000/QALY	**Results**: $44,000/QALY (PAD lay responder), $27,200/QALY (PAD police) **Author’s conclusions**: Training and equipping lay volunteers to defibrillate in public places may have an incremental cost-effectiveness that is similar to that of other common health interventions. **% EE assessment satisfied**: 52%
Ornato (1988)	**Perspective**: Not reported **Intervention/Comparator**: Basic EMT, EMTs trained in defibrillation, and paramedics **Simulated population**: Not applicable **Country/Currency (adj. year)**: USA;USD (NR)	**Modeling approach**: Not reported **Time horizon**: Not reported **Discounting**: n/a **Threshold used**: Not reported	**Results**: $7,687/LS basic EMT; $2,126/LS EMT trained defibrillation; $2,289/LS paramedics **Author’s conclusions**: From a medical and a cost-effective standpoint, all communities served by basic EMTs should consider upgrading them to at least the defibrillation trained EMT level. **% EE assessment satisfied**: 32%
Osorio Cuevas (2019)	**Perspective**: Health system **Intervention/Comparator**: CPR with AED vs basic CPR without the use of the defibrillator **Simulated population**: People with loss of consciousness in crowded spaces with large audiences. **Country/Currency (adj. year)**: Colombia;COP (2016)	**Modeling approach**: Decision-analytic model **Time horizon**: Unconsciousness to hospital admission **Discounting**: 5% **Threshold used**: 10 million COP/life saved	**Results**: 3,267,777 COP/ LS **Author’s conclusions**: A cardiopulmonary resuscitation program with early defibrillation using an AED in crowded public spaces is a cost-effective alternative for the Colombian Health System. **% EE assessment satisfied**: 80%
Perkins (2021)	**Perspective**: Health system **Intervention/Comparator**: Adrenaline **Simulated population**: 8014 Adults treated for an out-of-hospital cardiac arrest **Country/Currency (adj. year)**: UK;GBP (2016–2017)	**Modeling approach**: Decision-analytic model **Time horizon**: Lifetime **Discounting**: 3.5% **Threshold used**: £20,000 to £30,000/QALY	**Results**: £1,693,003 / QALY first 6 months or £81,070/QALY over lifetime **Author’s conclusions**: Adrenaline improved long-term survival, but there was no evidence that it significantly improved neurological outcomes. The incremental cost-effectiveness ratio per quality-adjusted life-year exceeds the threshold of £20,000–30,000 per quality-adjusted life-year usually supported by the NHS. **% EE assessment satisfied**: 84%
Rauner et al (2003)	**Perspective**: Societal **Intervention/Comparator**: AED programmes for the Austrian Red Cross/no AED program **Simulated population**: N/A **Country/Currency (adj. year)**: Austria;Euro (2003)	**Modeling approach**: Decision-analytic model **Time horizon**: Not reported **Discounting**: 3% **Threshold used**: $50,000/QALY	**Results**: €17,139/QALY **Author’s conclusions**: The decision of the Red Cross to equip all ambulances with AEDs was cost-effective. **% EE assessment satisfied**: 68%
Shaker (2022)	**Perspective**: Societal and Health system **Intervention/Comparator**: Portable SMall AED for Rapid Treatment of SCA (SMART) **Simulated population**: 600,000 sudden cardiac arrest risk patients who had not received implantable cardioverter defibrillator **Country/Currency (adj. year)**: USA;USD (2021)	**Modeling approach**: Markov model **Time horizon**: 50-year **Discounting**: 3% **Threshold used**: $100,000/ QALY	**Results**: Societal: $53,925/QALY; Healthcare: $59,672/QALY **Author’s conclusions**: A SMART approach to SCA prophylaxis prevents fatalities and is cost-effective in patients at elevated SCA risk. **% EE assessment satisfied**: 80%
Sharieff (2007)	**Perspective**: Payer **Intervention/Comparator**: On-site AED management compared with patients managed on-site without AEDs (Public access AEDs) **Simulated population**: Fictitious male and female new cardiac arresl patients in Ontario, Canada (mean age, 69 +- 13 years). **Country/Currency (adj. year)**: Canada;CAD (2005)	**Modeling approach**: Decision-analytic model **Time horizon**: 5 yrs **Discounting**: 3% **Threshold used**: Not reported	**Results**: $511,766 Office; $2,360,023 Apartment; $87,569 high risk homes; $1,529,371 homes of high risk > 55 **Author’s conclusions**: Indiscriminate deployment of AEDs is not a cost-effective means of improving health outcomes of cardiac arrest. Their use should be restricted to emergency response programs, high-risk sites (such as hospitals), and high-risk patients. **% EE assessment satisfied**: 84%
Shibahashi (2022)	**Perspective**: Payer **Intervention/Comparator**: BLS termination-of-resuscitation, ALS TOR or no TOR **Simulated population**: All-Japan Utstein registry of 126,271 patients median age 80 yrs **Country/Currency (adj. year)**: Japan;JPY (2013)	**Modeling approach**: Decision-analytic markov model **Time horizon**: Lifetime **Discounting**: 2% **Threshold used**: $45,455/ QALY	**Results**: $23,851/QALY **Author’s conclusions**: No-rule scenario was not cost-effective compared with BLS-rule scenario within acceptable willingness-to-pay thresholds. **% EE assessment satisfied**: 88%
Stokes (2021)	**Perspective**: Health system **Intervention/Comparator**: igel supraglotticairway (SGA) vs tracheal intubation (TI) **Simulated population**: 9296 non traumatic OHCA patients **Country/Currency (adj. year)**: UK;GBP (2016/17)	**Modeling approach**: Within trial economic analysis **Time horizon**: 6 months **Discounting**: No discount **Threshold used**: £20,000/ QALY	**Results**: No evidence of difference in cost-effectiveness **Author’s conclusions**: TI was more effective and less costly than igel; however differences were small and there was great uncertainty around these results. **% EE assessment satisfied**: 80%
Sund (2012)	**Perspective**: Not reported **Intervention/Comparator**: Fire stations in the County of Stockholm equipped with AEDs and dispatched in parallel with ambulances to all suspected cases of OHCA vs no dispatch of fire stations Simulated population: 836 patients with OHCA **Country/Currency (adj. year)**: Sweden;EUR (2007)	**Modeling approach**: Decision-analytic model **Time horizon**: 10 yrs **Discounting**: 4% **Threshold used**: €65,000/ life saved	**Results**: €13,000/QALY; €60,000/LS **Author’s conclusions**: The intervention of dual dispatch defibrillation by ambulance and fire services in the County of Stockholm had positive economic effects. The return on investment was high and the cost-effectiveness showed levels below the threshold value for economic efficiency used in **% EE assessment satisfied**: 72%
Urban et al. (1981)	**Perspective**: Not reported **Intervention/Comparator**: Paramedic program **Simulated population**: 1,035 patients **Country/Currency (adj. year)**: USA;USD (1978)	**Modeling approach**: Not reported **Time horizon**: 3 yrs **Discounting**: n/a **Threshold used**: $21,000/ LYS	**Results**: $42,358/LS **Author’s conclusions**: Even at the upper bound of cost per life saved, this program is cost-beneficial; it compares favorably with Acton’s estimate of the value of saving a myocardial infarction patient (approximately $48,000 in 1978 dollars). **% EE assessment satisfied**: 52%
Valenzuela (1990)	**Perspective**: Not reported **Intervention/Comparator**: EMS system capable of successfully resuscitating patients from OHCA vs. EMS system without this capability **Simulated population**: 190 patients experiencing nontraumatic, prehospital cardiopulmonary arrest in Tucson, Arizona, between October 1988 and July 1989. **Country/Currency (adj. year)**: USA;USD (1989)	**Modeling approach**: Not reported **Time horizon**: Not reported **Discounting**: 5% **Threshold used**: Not reported	**Results**: $118,939/ LS or $8,886/ LYS **Author’s conclusions**: Out-of-hospital treatment by paramedics of cardiopulmonary arrest is more cost effective than heart, liver, bone marrow transplantation, or curative chemotherapy for acute leukemia. **% EE assessment satisfied**: 60%
vanAlem (2004)	**Perspective**: Patient **Intervention/Comparator**: Reduction in time to shock of 2, 4, and 6 minutes. **Simulated population**: 308 patients OHCA patients witnessed with shockable rhythm **Country/Currency (adj. year)**: Netherlands;EUR (2001)	**Modeling approach**: Not reported **Time horizon**: 6 months **Discounting**: n/a **Threshold used**: €20,000/life saved	**Results**: €17,508/LS 2 min reduction ; €14,303/LS 4 min reduction; €12,708/LS 6 min reduction **Author’s conclusions**: Costs per survivor were lowest with the shortest time to shock because of shorter stay in the intensive care unit. Reducing the time to defibrillation increases the healthcare costs by an acceptable amount according to current standards and is economically attractive. **% EE assessment satisfied**: 64%
Vercammen (2020)	**Perspective**: Health system **Intervention/Comparator**: current OHCA care vs nation-wide Emergency Volunteer Application, a Belgian smartphone application that mobilizes volunteers to perform CPR and defibrillation with publicly available AED after an emergency call for suspected OHCA. implementation **Simulated population**: Patients suffering from witnessed OHCA of cardiac origin in the Belgian population (n = 6150 cases) **Country/Currency (adj. year)**: Belgium;EUR (NR)	**Modeling approach**: Accessible model **Time horizon**: 6 yrs **Discounting**: n/a **Threshold used**: Not reported	**Results**: €17,000/QALY **Author’s conclusions**: Nation-wide implementation of EVapp, a novel smartphone application to mobilize trained volunteers to nearby OHCA victims, would increase survival without major increase in costs. According to the best case estimates, the increase in survival for witnessed OHCA of cardiac origin was projected at 15% over the baseline scenario. This considerable increase in survival was not associated with a major increase in cost per QALY. **% EE assessment satisfied**: 56%
von Vopelius-Feldt (2019)	**Perspective**: Health system **Intervention/Comparator**: ALS vs prehospital critical care + ALS **Simulated population**: Adult non-traumatic OHCA **Country/Currency (adj. year)**: England;GBP (2016/2017)	**Modeling approach**: Markov model **Time horizon**: Lifetime **Discounting**: 3.5% **Threshold used**: £20,000/ QALY	**Results**: £11,407/QALY **Author’s conclusions**: While costs of either prehospital ALS and/or critical care per patient with OHCA are relatively low, significant costs are incurred during hospital treatment and after discharge in patients who survive. **% EE assessment satisfied**: 80%
Walker (2003)	**Perspective**: Health system **Intervention/Comparator**: Defibrillators in major airports, railway and bus stations/no public place defibrillators **Simulated population**: all prehospital cardiac arrests due to presumed heart disease that occurred in a major airport, railway, or bus station **Country/Currency (adj. year)**: Scotland;GBP (2000–2001)	**Modeling approach**: Not reported **Time horizon**: Lifetime **Discounting**: Outcomes 1.5%, Costs 6%. **Threshold used**: £30,000/ QALY	**Results**: £41,146/QALY **Author’s conclusions**: These costs represent poorer value for money than some alternative strategies, such as the use of other trained first responders, and exceed the commonly used cut-off levels for funding **% EE assessment satisfied**: 60%
Wei (2020)	**Perspective**: Health system **Intervention/Comparator**: Ambulance response time, bystander CPR and AED **Simulated population**: Not applicable **Country/Currency (adj. year)**: Singapore;SGD (NR)	**Modeling approach**: Decision-analytic model **Time horizon**: 1 yr **Discounting**: 3% **Threshold used**: Not reported	**Results**: Additional ambulances $13,6210/LYS; increased CPR training $29,5121/LYS; additional public AEDs $8,554/LYS **Author’s conclusions**: Investing in AEDs had the most gain in survival, compared with leasing additional ambulances or increasing the number of people trained in CPR. **% EE assessment satisfied**: 48%
Yen (2006)	**Perspective**: Health system **Intervention/Comparator**: EMT vs emergency physicians **Simulated population**: patients experiencing OHCA of non-traumatic origin with ALS activation, transported by EMS to nine medical centers in Taipei city, between November 1999 and December 2000 **Country/Currency (adj. year)**: Taiwan;USD (2000)	**Modeling approach**: Decision-analytic model **Time horizon**: n/a **Discounting**: No discount **Threshold used**: Not reported	**Results**: $21,136/ LYS **Author’s conclusions**: The use of EMTs as ALS care providers for OHCA patients in the two-tiered EMS system resulted in a reasonable cost-effectiveness ratio. EMTs could be considered as the second tier of EMS systems in urban areas in Taiwan. **% EE assessment satisfied**: 60%

**Abbreviations:** AED, automatic external defibrillator; ALS, advanced life support; COP, Colombian peso; CPR, cardiopulmonary resuscitation; EE, economic evaluation; EMS, emergency medical service; EMT, emergency medicine technician; GBP, Great British pound; HCM, hypertrophic cardiomyopathy; OHCA, out-of-hospital cardiac arrest; QALY, quality-adjusted life year; LTCF, long-term care facility; LYS, life years saved; SCD, sudden cardiac death; SGD, Singaporean dollar; USD, United States dollar; VF, ventricular fibrulation; VT, ventricular tachycardia.

**Table 2.** Incremental cost-effectiveness results of included studies by theme

Study	Location	Intervention	Findings per QALY or LY saved (currency standardized to 2022 USD)
**CPR and Bystander Training (n = 8)**			
Al-Badriyeh 2022^42^	Qatar	Out-of-hospital extracorporeal cardiopulmonary resuscitation (OH-ECPR) vs in-hospital extracorporeal cardiopulmonary resuscitation (IH-ECPR)	$127,634/ case of success
Bouland 2015^43^	US	Large scale community bystander CPR training	$27,012/QALY
Doan 2022^44^	Australia	Extracorporeal cardiopulmonary resuscitation (E-CPR) v conventional CPR (C-CPR)	$30,971/QALY
Groeneveld 2005^45^	US	CPR only vs CPR + AED (bystanders)	$298,727/QALY general population; $89,724/QALY if trainee lives w/ person > 75
Marti 2017^46^	UK	Mechanical CPR	Manual CPR dominates mechanical CPR
Nichol 2009^47^	US	CPR only vs CPR + AED (bystanders)	$78,185/QALY ; $48,333/QALY
Osorio Cuevas 2019^37^	Colombia	CPR only vs CPR + AED	$1,007/LS
Vercammen 2020^34^	Belgium	Smartphone application mobilizing CPR by bystanders	$20,534/QALY
**Increasing Access to Automated External Defibrillators (AEDs) (n = 18)**			
Andersen 2019^17^	US	Public Access AEDs	$15,582/QALY
Bauer 2021^39^	Germany	AED drone network	$27,749/LYS
Benson 2022^33^	England	School-based AEDs vs no AED usage	$11,398/QALY
Bogle 2019^48^	US	AED drone network	$11,584/QALY
Cram 2003^49^	US	Public Access AEDs	$46,493/QALY
Cram 2005^50^	US	In home AEDs to highrisk individuals	$134,280/QALY
Folke 2009^36^	Denmark	Public Access AEDs	$42,862/QALY (ERC) or $52,962/QALY (AHA)
Forrer 2002^51^	US	Police equipped with AEDs	$86,618/LS or $19,776/LYS
Foutz 2000^52^	US	AEDs in long term care facilities	$114,149/ LS
Groeneveld 2001^53^	US	AEDs on airlines	$59,074 on aircraft > 200 seats; $68,278 on aircraft > 100 seats; $158,479/QALY on all aircraft
Haag 2020^32^	US	In home AEDs to children with HCM	$94,285/QALY
Mears 2006^40^	US	Public Access AEDs– rural locations	$15,844/LS or $3,618/LYS
Moran 2015^38^	Ireland	Public Access AEDs	$114,798/QALY
Nichol 1998^54^	US	Public Access AEDs	$78,185/QALY
Nichol 2003^55^	US	Public Access AEDs	$85,913/QALY
Shaker 2022^18^	US	Portable SMall AED for Rapid Treatment of SCA (SMART)	Societal: $53,925/QALY; Healthcare: $59,672/QALY
Sharieff 2007^56^	Canada	Public Access AEDs	$539,434 Office; $2,487,615 Apartment; $92,303 high risk homes; $1,612,055 homes of high risk > 55
Walker 2003^57^	Scotland	AEDs (selectively placed)	$97,962/QALY
**Prehospital System Interventions (n = 19)**			
Achana 2020^28^	UK	Adrenaline use	$122,769/QALY
Aguilera Campos 2012^24^	Mexico	Increasing ambulances and training police in bystander CPR and AEDs to improve time to CPR/AED	$47,019 to 517,214 / life saved
Benger 2022^31^	England	igel supraglottic airway (SGA) vs tracheal intubation (TI)	Dominated
Cappato 2005^35^	Italy	AEDs added to ambulance system	$35,465/QALY
Ginsberg 2015^58^	Israel	Various prehospital interventions (BLS/ALS and ambulance transfer)	$35,776/DALY
Jakobsson 1987^22^	Sweden	Advanced EMT defibrillator training (8hr training program to distinguish ventricular tachycardia/ ventricular fibrillation from other rhythms and how to defibrillate expediently)	$36,713/LS
Jermyn 2000^59^	Canada	Equipping rural first responder fire departments with AED’s	$11,164/LS (urban; control), $81,183/LS (rural; experimental)
Nichol 1996^60^	Canada	Various prehospital interventions (improved response time/ambulance defibrillators)	$102,260/QALY
Ornato 1988^21^	US	Basic EMT vs EMT trained in defibrillation vs paramedics	$18,116/LS basic EMT; $5,010/ LS EMT trained defibrillation; $5,395/LS paramedic
Perkins 2021^29^	UK	Adrenaline use	$2,397,888/QALY first 6 months or $114,824/QALY over lifetime
Rauner 2003^61^	Austria	Equipping Austrian Red Cross Ambulances with AED’s	$25,840/QALY
Stokes 2021^30^	UK	igel supraglottic airway (SGA) vs tracheal intubation (TI)	No evidence of difference in cost-effectiveness
Sund 2012^25^	Sweden	Early defibrillation by dispatching AEDs with Fire as well as EMS	$18,662/QALY; $86,130/LS
Urban 1981^62^	US	Paramedic training/services	$184,320/LS
Valenzuela 1990^19^	US	Creation of EMS system for OHCA	$267,423/LS or $19,979/LYS
vanAlem 2004^26^	Netherlands	Reducing time to shock	$28,619/LS 2 min reduction; $23,381/LS 4 min reduction; $20,773/LS 6 min reduction
**Termination of resuscitation (n = 1)**			
Shibahashi 2022^63^	Japan	BLS termination-of-resuscitation (TOR), ALS TOR or no TOR	$23,851/QALY

## Data Availability

All data generated or analysed during this study are included in this published article and its supplementary information files

## Box 1: PubMed Search Terms

(“Out-of-Hospital Cardiac Arrest”[Mesh] OR “out of hospital cardiac arrest”[tw] OR “out of hospital cardiac arrests”[tw] OR “out of hospital heart arrest”[tw] OR “out of hospital heart arrests”[tw] OR “out of hospital cardiopulmonary arrest”[tw] OR “OHCA”[tw] OR “prehospital cardiopulmonary arrest”[tw] OR “pre-hospital cardiopulmonary arrest”[tw] OR “prehospital cardiac arrest”[tw] OR “pre-hospital cardiac arrest”[tw] OR “prehospital heart arrest”[tw] OR “pre-hospital heart arrest”[tw] OR “automated external defibrillator”[tw] OR “automated external defibrillators”[tw] OR “bystander cardiopulmonary resuscitation”[tw] OR “bystander CPR”[tw] OR “layperson cardiopulmonary resuscitation”[tw] OR “layperson CPR”[tw])

AND

(“Cost-Benefit Analysis”[Mesh] OR “cost benefit”[tw] OR “cost effective”[tw] OR “cost effectiveness” [tw] OR “economic evaluation”[tw] OR “economic evaluations”[tw] OR (“cost”[tw] AND (“benefit”[tw] OR “benefits”[tw])) OR “cost utility”[tw] OR “cost savings”[mh] OR “cost savings”[tw] OR “cost control”[tw] OR “costs and cost analysis”[mh:noexp] OR “cost analysis”[tw] OR “cost comparison”[tw])
